# Découverte tomodensitométrique d’un mésentère commun complet par perforation d’une appendicite aiguë

**DOI:** 10.11604/pamj.2017.27.3.11511

**Published:** 2017-05-02

**Authors:** Traore Abdoulaye Ababacar, Mvumbi Kutubisa Franck, Ly Seydou, Alaoui Lamrany Youssef, Boubbou Meryem, Maaroufi Maaroufi, Alami Badreeddine

**Affiliations:** 1 Service de Radiologie du CHU, Faculté de Médecine et de Pharmacie, Université Sidi Mohamed Ben Abdellah, Hassan II Fès, Maroc

**Keywords:** Adulte, mésentère commun complet, appendicite ectopique aiguë, TDM, laparotomie, Adult, complete common mesentery, ectopic acute appendicitis, CT scan, laparotomy

## Abstract

Le mésentère commun complet est une anomalie rare de rotation du tube digestif. Sa découverte est exceptionnelle à âgé adulte; à cette période, il demeure très souvent asymptomatique et donc non diagnostiquée. Le mésentère commun complet peut être découvert de façon fortuite, lors d’un syndrome appendiculaire ectopique, comme ce fut le cas de notre observation. Il s’agissait d’un patient âgé de 42 ans, qui à présenté un tableau inflammatoire clinico-biologique aiguë pelvien. La TDM a permis de poser le diagnostic d’une appendicite aigue sur un mésentère commun complet. Le traitement par laparotomie a confirmé le diagnostic d’une appendicite aiguë perforée sur la malrotation intestinale. Les suites post appendicectomies étaient favorables.

## Abstract (en)

Complete common mesentery is a rare abnormality in midgut rotation. Its detection is exceptional during adulthood because, during this period, is very often asymptomatic and therefore not diagnosed. Complete common mesentery may be discovered incidentally in patients with ectopic appendicular syndrome, as in our case study. We here report the case of a 42-year old patient presenting with acute clinico-biological pelvic inflammatory disorder. CT scan allowed the diagnosis of complete common mesentery associated with acute appendicitis. Laparotomy confirmed the diagnosis of perforated acute appendicitis associated with intestinal malrotation. Appendectomy outcomes were favorable.

## Introduction

L’appendicite aiguë est l’urgence chirurgicale abdominale la plus fréquente [1, 2]. Le diagnostic clinique de certitude d’une appendicite est difficile, en raison des nombreuses variations anatomiques qui peuvent être source de retard diagnostique et thérapeutique [1, 2]. La tomodensitométrie (TDM) occupe une place majeure pour le diagnostic des appendicites compliquées ou ectopiques. Elle permet un diagnostic anatomique, lésionnel précis et oriente la voie d’abord chirurgicale [3–7]. Nous apportons un cas supplémentaire de découverte tomodensitométrique d’une appendicite ectopique perforée sur un mésentère commun complet diagnostiqué à l'âge adulte.

## Patient et observation

**Figure 1:**
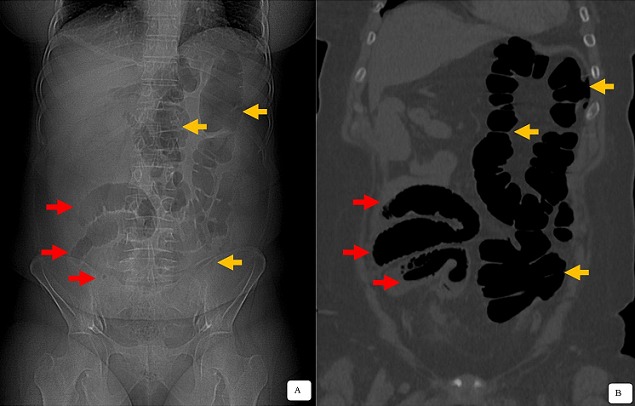
Cliché de scannogramme (A) et coupe TDM en reconstruction coronale (B) montre un mésentère commun complet avec des anses coliques gauches (flèche jaune) et gréliques droites (flèche rouge); appendicite ectopique pelvienne médio caecale (flèche bleu)

**Figure 2:**
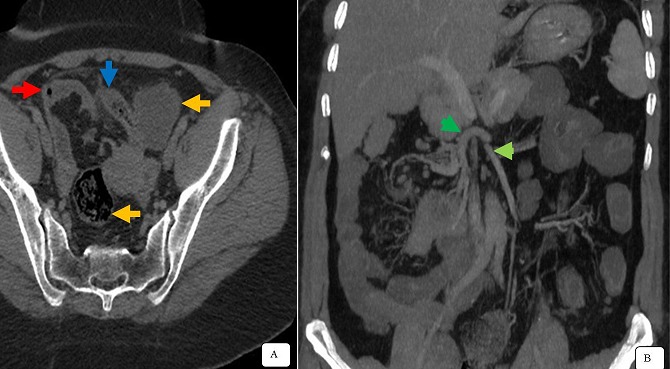
Coupes TDM axiale (A) et en reconstruction coronale (B) montre une appendicite aigue en situation ectopique pelvienne médio caecale (flèche bleu), sur un mésentère commun complet (flèches rouges et jaunes); infiltration de la graisse péri appendiculaire: les vaisseaux mésentériques étaient également transposés, artère (flèche verte) et veine (flèche verte)

Il s’agit d’un patient de 42 ans, diabétique sous antidiabétiques oraux depuis 4 ans. Il est admis au service des urgences pour un abdomen aigu. La symptomatologie remonte à 6 jours avant l’admission, par la survenu de douleurs pelviennes localisées puis devenant diffuses, associées à une hyperthermie chiffrée à 39°C. L’examen clinique trouve un patient conscient, tachycarde à 110 battements/minute, présentant une défense abdominale diffuse plus accentuée au niveau pelvien. Le bilan biologique de Numération Formule Sanguine montre un syndrome inflammatoire, avec une hyperleucocytose à polynucléaire neutrophile à 17,5 Giga/l, la C Réactive Protéine 320 mg/l et un taux d’hémoglobine normal à 12g/dl. L’échographie réalisée en première intention, retrouve une fosse iliaque gauche vide, sans autre anomalie spécifique décelée. Le complément TDM montre une appendicite aiguë en situation ectopique pelvienne médio-caecale, sur un mésentère commun complet avec des anses coliques gauches, et gréliques droites (Figure 1). Cette structure appendiculaire mesure 16mm de diamètre s’accompagnant d’une infiltration de la graisse tout autour ainsi des ganglions locorégionaux. Parallèlement, les vaisseaux mésentériques supérieurs étaient également transposés (Figure 2). La prise en charge chirurgicale en urgence par laparoscopie confirme un mésentère commun complet avec le cadre colique et le caecum pelvien à gauche, visualisation en médio-caecale d’un appendice phlegmoneuse et perforée à sa pointe. Le geste thérapeutique a consisté à la réalisation d’une appendicectomie après ligature et section du méso appendice. Les suites opératoires étaient simples sans complications. L’étude anatomo-pathologique de la pièce opératoire était une appendicite inflammatoire.

## Discussion

Les anomalies congénitales du tractus gastro-intestinal sont une cause importante de morbidité chez les enfants et moins fréquemment chez les adultes [2–5]. La prévalence de ces malformations congénitales est estimée à l'âge adulte de l'ordre de 0,2% à 0,5% [2, 5]. Au cour du développement embryologique, l’intestin subit des phénomènes complexes de réintégration, rotation et accolement. Lorsque ces phénomènes sont incomplets ou vicieux, ils peuvent aboutir à des situations anatomiques potentiellement pathologiques [2, 5]. En occurrence, les anomalies de migration du mésentère: absence totale de rotation, mésentère commun complet, mésentère commun incomplet et rotation inverse, en cas de situs inversus [7]. Embryologiquement, la première rotation se déroule avant la 10^ème^ semaine de gestation lorsque l’intestin primitif est encore situé hors de l’abdomen. Cette rotation place la portion prévitelline (grêle) à droite et la portion post vitelline (colon) à gauche; un arrêt à ce stade est à l’origine du mésentère commun complet [8]. Le mésentère commun complet résulte alors d’un arrêt de la rotation intestinale à 90°. Ainsi se situe, le cadre colique à gauche et intestin grêle à droite; le caecum en position antérieure et médiane et l’artère mésentérique supérieure à droite de la veine mésentérique supérieure; et l’isomérisme pulmonaire gauche [7].

Les malformations intestinales demeurent très souvent asymptomatiques et donc non diagnostiquées à l’âge adulte [8]. A cet âge, le mésentère commun complet est souvent découvert, soit fortuitement, soit dans le cadre d’une pathologie tumorale ou inflammatoire du tube digestif, notamment d'une appendicite ectopique dont le diagnostic clinique a souvent erré voir été non évoqué [4, 5, 7, 8]. Le mode de découverte de notre patient a été fortuit suite à un syndrome inflammatoire aigue pelvien, comme ce fut également le cas dans d’autres observations [5, 6]; chez Aziz El Madi et al, l’âge de découverte était par contre plus jeune plus jeune. Par conséquent, la localisation inhabituelle des appendicites ectopiques est responsable d’un retard de diagnostic pouvant être à l’origine de complications graves [7, 8]. Le diagnostic d’appendicite sur malrotation intestinale se fait de plus en plus précocement, grâce à l’imagerie moderne [7]. L'abdomen sans préparation peut être extrêmement variable et ne montre aucun signe spécifique, cependant il est rarement normal et généralement interpréter comme « inhabituel » ou discordant [2, 3]. Le lavement baryté a permis d’évoquer le diagnostic de mésentère commun complet dans l’observation Aziz EL Madi et al [6]. L'échographie doppler est souvent gênée par les gaz et n'est pas toujours contributive au diagnostic [7]. Selon certains auteurs, l'échographie serait l'examen de référence pour éliminer une malrotation intestinale, lorsque celle-ci montre la présence du troisième duodénum en arrière de l'artère mésentérique supérieure [7]. L’échographie était non spécifique chez notre patient, ainsi que dans d’autres observations [5, 6].

L’apport de la TDM apparait donc essentiel pour redresser le diagnostic, mais aussi de manière générale dans les syndromes douloureux abdominaux atypiques [3, 7]. Elle est supérieure à l’échographie pour les appendicites compliquées ou ectopiques, essentiellement chez les sujets de plus de 40 ans [5]. Elle a permis, dans notre cas, de confirmer le diagnostic d’appendicite aigue sur mésentère commun complet; comme ce fut le cas dans d’autres observations [5, 6]. Dans l’observation de J Flesch et al [5], des bulles d’air péri appendiculaires vues témoignant d’une perforation, élément qui n’a pas été observé chez notre patient. En plus, la TDM abdominale permet d’orienter la voie d’abord chirurgicale [3]. La laparoscopie est primordiale dans ces cas puisqu’elle permet de confirmer l’anomalie anatomique, puis proposer un traitement qui consisterait à une appendicectomie sous cœlioscopie [9, 10]. Ce fut la stratégie de pris en charge de notre patient, par contre les observations de J Flesch et al [5] ainsi que de Aziz El Madi et al [6] ont été opéré avec succès par cœlioscopie. L’évolution post opératoire de notre observation était rapidement favorable sans complication.

## Conclusion

La présentation atypique d’une appendicite aiguë constitue un défi diagnostic en urgence. Ce tableau clinique doit évoquer, l’éventualité d’une appendicite ectopique associée à une malformation intestinale, en occurrence le mésentère commun complet. L’imagerie, essentiellement la TDM permet d’établir le diagnostic et de préciser les variations congénitales du tube digestif, à fin de guider la prise en charge chirurgicale d’urgence.

## Conflits d’intérêts

Les auteurs ne déclarent aucun conflit d'intérêt.
